# Supplementary material for: Women empowerment domains and unmet need for contraception among married and cohabiting fecund women in Sub-Saharan Africa: A multilevel analysis based on gender role framework
Source: PLoS One. 2023 Sep 8;18(9):e0291110. doi: 10.1371/journal.pone.0291110 (PMC10491392; doi:10.1371/journal.pone.0291110)
Supplement: Attachment — Submitted filename: review.docx [file pone.0291110.s001.docx]

Note to Editor:

Sending a pdf file to the reviewer made the review difficult. I could not directly insert comments as I would in a doc file. Hence, I am making no recommendation.

Women empowerment indices and unmet need for contraception among Married women in Sub-Saharan Africa: A multilevel analysis based on Gender Role Framework

Abstract

Background

Low women empowerment status, is a known contributing factor to unmet needs for contraception by limiting access to health service delivery points through negative cultural beliefs and practices. Although the unmet need for birth control methods continues to be a major challenge in Sub-Saharan African countries (SSA) countries, little is known about the relationship between unmet needs and domains of women empowerment in the region.  Hence, this study aimed at assessing the influence of women empowerment indices on unmet need for contraception in the 18 SSA countries with the most recent DHS report (2016-2021).

Methods

The appended women (IR) file of the most recent Demographic and Health Survey data for SSA nations from 2016–2021 provided the data for this study. A total of 127,650 (with a weighted frequency of 128,939) married women were included and analyzed by using STATA version 16. The Harvard Institute's Gender Roles Framework, which comprised three domains namely, influencing, resource, and decision-making, was employed to identify and categorize the women empowerment indices. The effects of each predictor on the unmet need for spacing and limiting were examined using a multivariable multilevel mixed-effect multinomial logistic regression analysis to consider data clustering. Adjusted relative risk ratio (aRRR) with its corresponding 95% confidence interval was used to declare the statistical significance of the independent variables

Introduction

In developing countries, more than 200 million women who wish to postpone or avoid pregnancy do not use family planning[1]. Rapid population growth in Sub-Saharan African (SSA) region is expected to continue until the end of the twenty-first century[2]. This region contains nearly all of the countries where fertility rates exceed five children per woman and where fertility rates are declining slowly[3, 4]. Promoting access to family planning in SSA regions continues to be a significant barrier to women's reproductive health, according to the Family Planning 2020 (FP2020) report[5]. Meanwhile, the proportion of unmet need for contraception among married women aged 15-49 in SSA exceeds 25% (i.e. about 47 million), compared to 14% in East Asia and 28% in Latin America and the Caribbean[6]. Unmet needs among these women can lead to unwanted pregnancies, unsafe abortions, ill health, and financial hardship for families and society[1]. Access to contraceptives has been noted as one of the ways to help reduce maternal mortality[7, 8]. Despite the government's efforts to make contraceptive services more accessible and affordable, inequalities in contraceptive acceptance rates have been high in SSA, resulting in higher unmet needs [14]. A woman is considered to have an unmet need for FP if she desires to stop or delay/postpone childbearing but does not use any method of contraception[9]. Unmet need for contraception is a significant indicator of the discrepancy between women's intentions for having children and their acceptance of contraception. It also measures how far the nations have come toward achieving universal access to sexual and reproductive health services[10]. Unmet needs for spacing and limiting are the two main categories. An unmet need for spacing is when a woman wants to delay or postpone getting pregnant, whereas an unmet need for limiting is when she wants to have no more children and is not using any form of contraception[9]. This unmet need accounted for the majority (82%) of unintended pregnancies and related unsafe abortions and delayed or no prenatal care [11]. And if all of the demand for contraceptives is met, the number of unintended pregnancies in developing countries, including in the SSA subregion, would drop from 75 million to 22 million annually[9]. With this, there 4 would be 22 million, 15 million, and 90,000 fewer unintended pregnancies, unsafe abortions, and maternal deaths respectively[12, 13]. All these can be achieved by strengthening initiatives that empower women. One dimension that has been shown to influence contraceptive use is women empowerment. Since the launch of the International Conference on Population and Development (ICPD) in Cairo in 1994, women empowerment has become a focus in the population sector[14]. According to the World Bank, Women empowerment is "the process of increasing a woman's or women’s capacity to make meaningful choices and transform them into desired behaviors and results[15]. Others define women empowerment as having the ability to act, independence, involvement, self-direction, consciousness, freedom, movement, and self-confidence [16, 17]. Women's empowerment has become a cornerstone for societal development so it adds value to the creation of more effective, knowledgeable, peaceful, and prosperous societies[18]. Women empowerment has the potential to increase contraceptive use[19, 20]. Low women empowerment status, on the other hand, is a known contributing factor to unmet needs for contraceptives by limiting access to health service delivery points through negative cultural beliefs and practices [21, 22]. In some parts of the SSA, patriarchal rule impairs women's ability to exercise their fundamental reproductive health rights[23]. Furthermore, some family planning initiatives in SSA failed to achieve their intended goal because they failed to take into account the power dynamics between women and their partners[24, 25]. Women in this region are unable to make decisions about whether or not to use contraception, as well as the type of contraception to use[26]. Studies in lower- and middle-income countries have identified an association between unmet needs and poor decision-making abilities in women, low media exposure, a lack of formal education, a high attitude towards wife beating, and unemployment[18, 27, 28]. The Harvard Analytical Framework (gender roles framework) developed by the Harvard Institute for International Development categorizes women empowerment indices into three dimensions: influencer, resource, and decision-making factors[29]. Influencing factors include variables that are symbolic of gender norms and beliefs, such as the gender division of labor, access to, and individual factors [30]. Resource factors include human capital and access to resources, whereas decision-making factors include women's participation in decision-making, including access to and use of resources[29, 30]. 5 Estimates of unmet needs are still a crucial indicator of the success of family planning programs and the amount of demand for contraception in developing nations, but there are some concerns about the precision of these estimates. Although the unmet need for contraceptive methods continues to be a major challenge in SSA countries, little is known about the relationship between unmet needs and domains of women empowerment in the region. So, using a gender analysis framework created by the Harvard Institute, this study looked into the relationship between women's empowerment indicators and unmet contraceptive needs in 18 SSA countries to make useful recommendations

Methods Data source, population, and study period A total of 18 demographic and health surveys (DHS) carried out in SSA countries between 2016 and 2021 were considered in this study. The DHS is conducted in 90 countries to collect information on fundamental health indicators. The data for this study came from the appended women's (IR) file, which contains information about contraception, and all important covariates. The study included countries with recent standardized DHS reports from 2016 to 2020 that had complete cases on the relevant variables. Only married, reproductive-age women who were exposed to frequent sexual activity were included. In contrast, infecund women were not supposed to use contraceptives and were excluded from the dataset before analysis[31]. The entire analysis relied on a weighted sample of 128,939 married and fecund women (Table

Why is the focus on married women alone? How about cohabiting, divorced/separated women who are also sexually active?

Why did the authors report significant p-value <0.20?

Why would a confidence interval “0.95(0.89, 1.01)” be significant? See Table 7c

The use of too many explanatory variables make the results difficult to follow

The discussion needs a significant improvement. Being a study that focused on many countries where data were collected in different years, it should be stated that the comparisons made could be misleading
